# Mechanisms underlying acid tolerance in *Lysinibacillus sp.* ND7, a highly efficient nitrate degrader

**DOI:** 10.1371/journal.pone.0336631

**Published:** 2025-11-13

**Authors:** Feifei Wang, Min Ai, Xiafei Yin, Lixue Liu, Hefei Shi, Guobin Liang

**Affiliations:** 1 School of Resources and Environmental Engineering, Jiangsu University of Technology, Changzhou, P. R. China; 2 Consulting Department, Jiangsu Longheng Environmental Technology Co., LTD, Changzhou, P. R. China; 3 School of Chemistry and Chemical Engineering, Jiangsu University of Technology, Changzhou, P. R. China; South China University of Technology, CHINA

## Abstract

In this study, the acid-tolerant strain *Lysinibacillus sp.* ND7 demonstrated excellent nitrate removal efficiency under acidic conditions, achieving a nitrate removal rate of 80.70 ± 0.02% in wastewater at pH 3.1. Metabolomics analysis revealed significant enrichment in pathways related to starch and sucrose metabolism, teichoic acid biosynthesis, and the phosphoenolpyruvate-dependent sugar phosphotransferase system (PTS). These pathways play a crucial role in providing energy, regulating metabolism, and maintaining cellular stability during nitrate degradation. Proteomic analysis showed a significant up-regulation of genes involved in the nitrate removal pathway. Additionally, the up-regulation of ABC transporter protein genes and the down-regulation of genes involved in oxidative phosphorylation and electron transport worked together to support the nitrate metabolic pathway and help the strain resist acid stress. In conclusion, these findings underscore the impressive nitrate removal capacity of the novel strain ND7 and offer new insights into the mechanisms through which ND7 resists acid stress under acidic conditions.

## 1. Introduction

Nitrate, when present in high concentrations in the environment, is recognized as a major macro-pollutant. While nitrate naturally occurs in low concentrations, human activities have introduced artificial sources, including the use of synthetic fertilizers, industrial wastewater, and domestic sewage discharges [1]. With the rapid pace of industrialization, acid washing technology has become widely used in metal processing, chemical production, and electronic manufacturing. Among various industrial wastewaters, acid washing wastewater, especially that containing high concentrations of nitrate, has garnered significant attention. This type of wastewater is generated during the industrial acid washing process, where metal surfaces are treated with nitric acid (HNO_3_) or other acidic solutions containing nitrate. Typically, it has a high nitrate concentration and is strongly acidic, with a low pH. Nitrate contamination has emerged as one of the most pressing water quality issues worldwide [2]. If not properly treated, nitrate-laden acid washing wastewater can contaminate groundwater and drinking water, posing serious risks to both the environment and human health [3]. Nitrates accumulate in water bodies, contributing to eutrophication, reducing the water’s self-purification capacity, and decreasing dissolved oxygen levels, which can lead to the death of aquatic organisms [4]. Beyond environmental concerns, the potential harm of nitrates to human health is also alarming. When nitrate levels are elevated, some nitrate is converted into harmful metabolites, such as nitrite ions, through bacterial reduction during food processing or intestinal transit. High nitrate concentrations in drinking water can impair the ability of human erythrocytes to carry oxygen, leading to methemoglobinemia, especially in children [5]. Nitrates may also contribute to the formation of carcinogenic compounds, such as N-nitrosamines and N-nitrosamides [3]. Given these challenges, developing low-cost and high-efficiency technologies for treating high-nitrate wastewater is crucial for environmental protection and ecological restoration.

Biological treatment has become the most widely used technology for industrial wastewater treatment due to its economic advantages, and it has been shown to effectively degrade nitrate [6]. Biological denitrification, a key process in wastewater denitrification, converts nitrate into nitrogen gas (N₂). Denitrification occurs with the assistance of functional heterotrophic denitrifiers under anaerobic conditions [7]. The process involves several key enzymes: nitrate reductase (*Nar*) reduces nitrate to nitrite, nitrite reductase (*Nir*) converts nitrite to nitric oxide (NO), and NO reductase (*Nor*) further reduces NO to nitrous oxide (N₂O). Finally, N₂O is converted to N₂ through the action of N₂O reductase (*Nos*) [8]. Despite the advantages of biological denitrification, two major challenges remain in treating high-nitrate acid-washing wastewater. Firstly, the relatively high concentrations of substrates, including nitrate and other components, can inhibit bacterial activity [9]. As a result, it is often necessary to isolate and select specialized bacteria that are adapted to the specific conditions of the wastewater. Secondly, the accumulation of nitrate in acid-washing wastewater can lead to self-inhibition of the degradation process, particularly under low pH conditions [10]. Previously, research on the biological treatment of nitrate mainly focused on model strains under neutral pH conditions, such as *Pseudomonas aeruginosa* DM6 (pH 8.0) [11], these studies have successfully revealed the key role of denitrification pathways in neutral environments. However, the adaptability mechanism and degradation potential of microorganisms in the harsh environment of acidic wastewater (pH < 5.0) are still far from clear. Therefore, in-depth research into the mechanisms of acid stress resistance at high nitrate concentrations could provide new insights into bacterial nitrate removal at the genetic level and offer a theoretical foundation for the targeted modification of microorganisms through genetic engineering.

Current research on nitrate biodegradation has primarily focused on nitrogen removal and its metabolic pathways. However, there has been limited exploration of the isolation and characterization of strains capable of degrading acidic wastewater. To date, only a few strain have been identified for the degradation of nitrate in acidic wastewater, and the metabolic mechanisms involved in this process remain unclear. Under extreme environmental conditions, such as low pH, many bacteria enter a viable but non-culturable (VBNC) state, which significantly hampers their metabolic activity and overall functionality [12]. The VBNC state represents a significant challenge for wastewater treatment and other biotechnological applications, as bacterial performance can be severely compromised [13]. Therefore, further research is needed to screen for efficient, acid-tolerant nitrate-degrading bacterium that can adjust to pH variations and to investigate their nutrient removal characteristics and transformation pathways.

In this study, a novel nitrate-degrading bacterium was first isolated from activated sludge. Subsequently, the effect of different strain doses on nitrate removal under low pH conditions was investigated. Metabolomic and proteomic analyses were then employed to explore the metabolic mechanisms of nitrogen transformation and the pathways involved in nutrient conversion. Crucially, multi-omics evidence indicates that ND7 primarily removes nitrate through assimilation pathways rather than classical denitrification, establishing it as a novel model for nitrate removal in acidic environments. The findings of this study will provide a scientific foundation for the development of efficient biological treatment technologies, offering new ideas and approaches for further research in industrial wastewater treatment.

## 2. Materials and methods

### 2.1. Culture medium and reagents

Luria–Bertani (LB) medium is included (per liter): 10 g Tryptone, 5 g Yeast extract, 10 g NaCl. Agar powder, H_2_SO_4_, HCl, and NaOH were purchased from Sinopharm Chemical Reagent Co., Ltd. (Shanghai, China). All reagents utilized were of analytical grade.

### 2.2. Isolation and identification

Isolation of a novel nitrate-degrading strain (ND7) from activated sludge of a wastewater treatment plant by gradient dilution and plate scribing [14]. The morphology of the ND7 was observed through a microscope (Olympus CX33, Japan). The 16S rDNA gene was amplified by polymerase chain reaction (PCR) using universal primers F27 and R1492, and sequenced by Shanghai Sangon Company. Sequence alignment was conducted in GenBank by BLAST (https://blast.ncbi.nlm.nih.gov/Blast.cgi). A neighbor-joining phylogenetic tree was constructed using MEGA 11.0 software. Average Nucleotide Identity (ANI) values were calculated using the OrthoANI algorithm, implemented through the Orthologous Average Nucleotide Identity Tool (OAT) [15], to assess the genome sequence-based similarity between ND7 and other reference strains [16].

### 2.3. Experimental methodology

The acid washing wastewater in this experiment comes from a factory in Changzhou. The wastewater contains a large amount of organic and inorganic pollutants, such as heavy metals (Fe^3+^, Cu^2+^), salts (nitrates), etc. The pH is below 1 and the nitrate content is 31200 ± 252 mg/L.

The strain ND7 was inoculated at 2% v/v into 100 mL of LB medium and cultured to a logarithmic phase of growth. The bacterial solution was centrifuged to separate the strain from the liquid, and the resulting strain were then placed into 100 mL of wastewater diluted 2000 times for biodegradation (pH 3.1, nitrate 15.6 ± 0.1 mg/L). Anaerobic incubation was performed for 120 h. Regularly take samples to determine the content of TN, NO_3_^−^-N, NO_2_^−^-N, and NH_4_^+^-N during incubation. All the above experiments are conducted in triplicate.

### 2.4. Analytical methods

The changes in the concentrations of Nitrates (NO_3_^-^-N) and Total Nitrogen (TN) were measured according to the Chinese State Environment Protection Agency [17]. A microplate reader (Infinite 200PRO, Beijing Longyue Biotechnology Development Co., Ltd., Beijing, China) was used to measure OD_600_ value. Differential metabolites identified by LC-MS and associated with different pathways were visualized by heatmapping and metabolomics pathway analysis. For each condition, samples were prepared from three independent biological replicates (n = 3). Statistical significance was determined using a t-test with an FDR-adjusted p-value < 0.05. Volcano plot analysis and metabolic pathway analysis were performed using MetaboAnalyst 5.0 [18]. The Kyoto Encyclopedia of Genes and Genomes (KEGG) database (http://www.kegg.jp/) was used to further characterize the location and function of these metabolites in various metabolic pathways [19,20].

## 3. Results and discussion

### 3.1. A novel nitrate degrading bacterium ND7 and its degradation characteristics

#### 3.1.1. Isolation and characterization of ND7.

**Fig 1:**
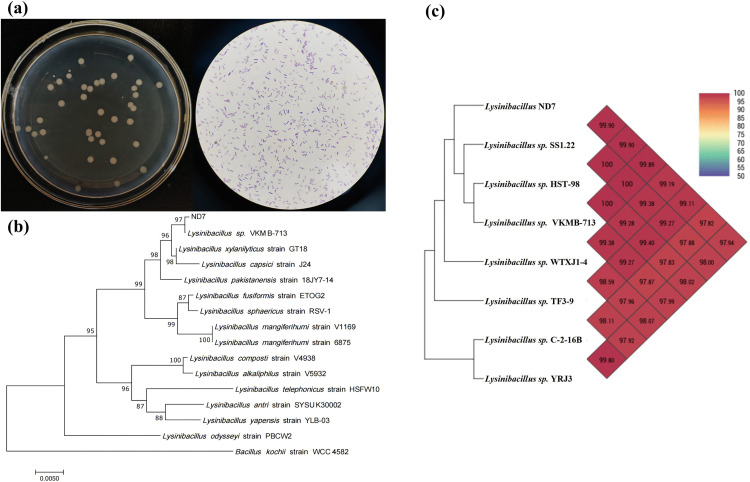
(a) Observation of the morphology of ND7. (b) Phylogenetic tree based on neighbor linkage analysis of partial 16S rDNA gene sequences. (c) ANI values between strain ND7 and other reference strains.

In the sludge, a highly effective NO_3_^-^-N-degrading strain with a degradation ratio of more than 80% was screened and named ND7. The colony morphology of ND7 on LB solid medium is shown on the left side of Fig 1a. The colonies were yellowish in color, with a moist, opaque surface and well-defined edges. The Gram staining method was used to observe ND7 under an optical microscope after staining [21]. As shown on the right side of Fig 1a, the strain appears purple, indicating that ND7 is a Gram-positive bacterium. Under light microscopy, the strain appeared as short, rod-shaped cells. The 16S rDNA gene was amplified by PCR using the universal primers F27 and R1492, and the resulting sequence was sent for sequencing by Shanghai Sangon Company. Sequence alignment was performed using BLAST on GenBank. A neighbor-joining phylogenetic tree was constructed using the MEGA 11.0 software. The phylogenetic tree of the strain is shown in Fig 1b, indicating that the obtained strain is *Lysinibacillus sp*. BLAST analysis of the 16S rDNA sequences of strain ND7 was performed by comparing it with other 16S rDNA sequences in GenBank. The ANI calculation method was then used to analyze the genomic correlation between strain ND7 and its seven nearest strains (Fig 1c). It is widely believed that the ANI value of the same species is greater than 95% [22]. This study found that the similarity in genetic sequences between ND7 and *Lysinibacillus sp.* was close to 99.90%, so ND7 was preliminarily identified as *Lysinibacillus sp.* [23]. Bacterium ND7 was stored in the China Center for Type Culture Collection (CCTCC No: 28761).

#### 3.1.2. Degradation characteristics of DN7 on high nitrate pickling wastewater.

**Fig 2:**
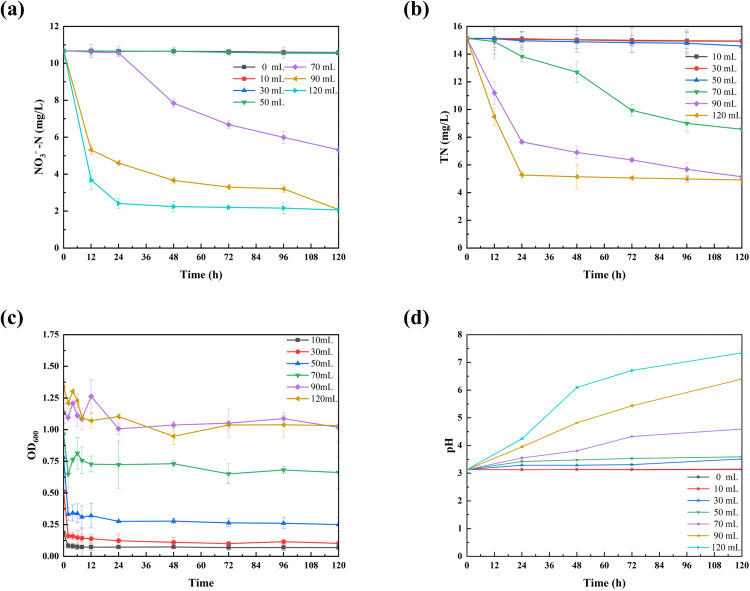
Changes in NO_3_^-^-N content (a), TN content (b), OD_600_ (c), and pH (d) of wastewater with different bacterial strains dosage.

The strain ND7 was inoculated into LB medium and cultured until the OD_600_ reached the end of the logarithmic growth phase. Then, 10 mL, 30 mL, 50 mL, 70 mL, 90 mL, and 120 mL of the bacterial solution were centrifuged and added to 100 mL of wastewater. The changes in NO_3_^-^-N, TN, OD_600_, and pH of the wastewater during the degradation process are shown in Fig 2. It’s clear that as the reaction time increases, NO_3_^-^-N in the wastewater was hardly degraded when the strain DN7 dosage was 10 mL, 30 mL, or 50 mL. This may be because strain ND7 did not survive in the wastewater. As shown in Fig 2c, the OD_600_ values decreased significantly after ND7 was added to the wastewater, indicating that the small number of strains added were unable to grow. This could be due to the low pH, which likely hindered the growth of the small inoculum [24]. When the dosage was 70 mL, 90 mL, and 120 mL, the degradation efficiency of NO_3_^-^-N reached 50.20 ± 0.20%, 80.50 ± 0.33%, and 80.70 ± 0.02%, respectively, after 120 hours of degradation. The higher dosages were more favorable for improving the degradation rate of NO_3_^-^-N in wastewater. This may be because the strains survived at higher dosages, with OD_600_ values exceeding 0.5 (Fig 2c), indicating good growth and metabolic activity under conditions of higher strain concentrations. Higher strain concentrations can improve the survival and metabolic activity of the strains. This is because, at higher concentrations, the strains are able to utilize the available resources in the medium more efficiently, leading to increased metabolic activity. For example, researchers such as Wang et al. [25] found that higher biomass concentrations were better equipped to adapt to environmental stresses and exhibited enhanced metabolic activity. Specifically, strain at higher densities can boost their metabolic activity through quorum sensing or by more effectively utilizing available nutrients [26].

Interestingly, when the inoculation volume was 70 mL, no significant degradation occurred within the first 24 hours. This could be due to the relatively low inoculation volume of 70 mL, which may have resulted in the microorganisms being in a less active state during the initial stages, thus delaying the degradation process. However, with inoculum volumes of 90 mL and 120 mL, the nitrate reduction rates reached 50.26% and 65.55%, respectively, within the first 12 hours. Over the next 12 hours, the nitrate concentration continued to decrease, with the 120 mL inoculum achieving a nitrate degradation efficiency of 77.39%, after which it stabilized. In contrast, with a 90 mL inoculum, nitrate degradation efficiency steadily decreased over 120 hours, reaching 80.50 ± 0.33% by the 120th hour. These results clearly indicate that increasing the inoculum volume significantly enhances the nitrate (NO_3_^-^-N) degradation efficiency. It was observed that after the 48th hour, the concentration of NO_2_^-^-N reached 0 (S1 Fig), and the TN concentration also decreased (Fig 2b). The changes in the pH of the wastewater further confirmed the occurrence of the NO_3_^-^-N degradation (Fig 2d). The pH of the wastewater reached 6.40 ± 0.04 and 7.34 ± 0.05 at inoculum dosages of 90 mL and 120 mL, respectively, after 120 hours. The reduction of nitrate and nitriteconsumes H^+^ ions, which means that as nitrate is degraded, the concentration of H^+^ in the solution decreases, resulting in an increase in pH. When the inoculum dosage increases, more strain are involved in the nitrate degradation process, accelerating the nitrate degradation rate. As more nitrate is reduced, more H^+^ ions are consumed, leading to an increase in pH [27]. The results of this study indicate that during the treatment process with ND7, nitrite is only briefly present as an intermediate product, with its concentration consistently remaining below 0.6 mg/L. Although ammonium salts accumulate, as shown in S2 Fig, their concentration stays below 0.1 mg/L, and the peak concentration is significantly lower than the known half-effective concentration (EC50) for common aquatic organisms [28], such as algae and water fleas. These new data suggest that the metabolic pathway of ND7 is highly efficient, with minimal accumulation of intermediate products. Concurrently, non-biological control experiments showed no significant change in nitrate concentration within the sterilized control group (S3 Fig), confirming that nitrate removal is primarily driven by the biological activity of ND7.

As shown in Table 1, the NO_3_^-^-N removal efficiency of ND7 (80.70 ± 0.02%) in this study was higher than that of *Bacillus simplex* H-b (67.29%) reported by Yang et al. [29]. Additionally, ND7 exhibited more stable removal performance at 35°C, maintaining a efficiency of 80.70 ± 0.02% over 120 hours. In contrast, the removal efficiency of *Pseudomonas stutzeri* YG-24 [30] was lower, at only 70.83%, while ND7 showed higher NO₃^-^-N degradation efficiency under similar conditions. Furthermore, ND7 also outperformed *Acinetobacter sp.* JR1 (68.3%), as reported by Yang et al. [31], which exhibited lower removal efficiency. Although *Comamonas sp.* pw-6 achieved a high NO₃^-^-N removal efficiency of 89.84% in the study by Chen et al. [11], its reaction requires succinate as a carbon source, which could result in higher energy and resource consumption. Therefore, the findings of this study suggest that optimizing strain dosing could effectively enhance NO₃^-^-N degradation efficiency.

**Table 1:** Efficiency of NO_3_^-^-N-containing wastewater degradation by different bacterial strains.

Bacteria	NO₃^-^-N concentration of initial wastewater (mg/L)	Degradation conditions	Degradation efficiency	References
*Bacillus simplex* H-b	63.0	168 h, 10°C	67.29%	[29]
*Pseudomonas stutzeri* YG-24	100	50 h, 25 ~ 35°C, 150 rpm	70.83%	[30]
*Acinetobacter sp.* JR1	51.6	48 h, 30°C, 120 rpm	68.3%	[31]
*Comamonas sp.* pw-6	150	24 h, 25°C, 160 rpm	89.84%	[11]
*Lysinibacillus sp.* ND7	15.6 ± 0.1	120 h, 35°C	80.70 ± 0.02%	This study

Although Table 1 summarizes the performance of various strains in this field, it is important to note that the data were obtained under different experimental conditions, such as temperature, time, and rotational speed. These factors significantly impact the metabolic activity and final product yield of the strains [32]. Therefore, the data presented in the table should not be compared directly or quantitatively across strains, but rather should be used as a reference for a performance range. The advantage of the ND7 strain in this study is primarily seen in its ability to achieve a competitive performance level under relatively specific conditions compared to other strains reported in the literature, which suggests potential for future industrial applications.

### 3.2. Metabolomic analysis

In the nitrogen metabolism pathway, nitrate can be degraded through two main processes. The first pathway involves denitrification, where nitrate is initially reduced to nitrite and then further converted into N_2_. The second pathway reduces nitrate to nitrite, followed by the reduction of nitrite to ammonia. The ammonia produced is then utilized for the synthesis of amino acids, which support microbial growth and metabolism.

**Fig 3:**
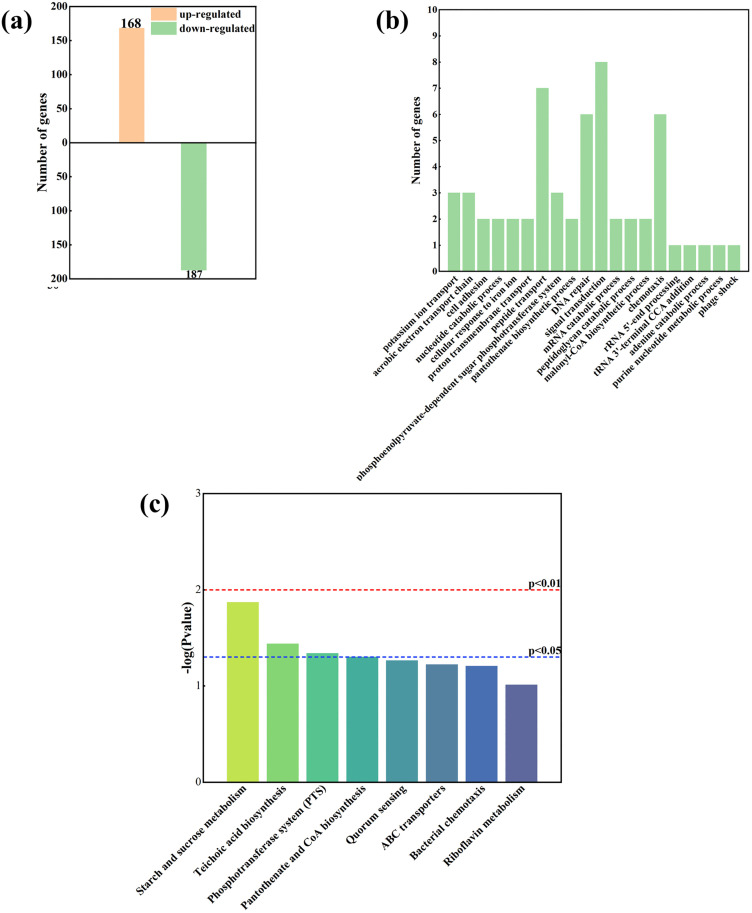
(a) KEGG pathway enrichment analysis (p < 0.05). (b) Volcano plot (p < 0.05). (c) Pathways of Nitrogen Metabolism.

Based on the KEGG [33] enrichment analysis results of differentially expressed metabolites, the top 30 pathways with the smallest p-values—representing the most significantly enriched pathways—were selected and visualized in a bar graph (Fig 3a). As shown in Fig 3a, the biosynthetic pathway of amino acids was notably significant during the degradation process, suggesting that nitrate was most likely assimilated into amino acids.

To investigate the degradation mechanism of ND7 under acid stress, we compared the metabolic differentiators before and after degradation. As shown in Fig 3b, a total of 347 metabolites were significantly altered. Among these, several were notably upregulated, including Aspartyl-L-proline, Glycylglycine, N-Acetyl-L-glutamic acid, and L-Glutamic acid. These findings suggest the involvement of specific metabolic pathways in stress response and degradation processes, potentially offering insights into acid-induced degradation mechanisms of ND7. Aspartyl-L-proline, a potential intermediate in proline metabolism, may act as a carbon source for the tricarboxylic acid (TCA) cycle. Its upregulation indicates that proline and its derivatives may function as osmoregulatory compounds, helping microorganisms maintain osmotic balance under high-nitrate conditions. Glycylglycine, a glycine derivative, serves as a precursor in amino acid biosynthesis and plays a key role in nitrogen metabolism, particularly in purine and pyrimidine synthesis. N-Acetyl-L-glutamic acid (NAG) is involved in the biosynthesis of amino acids such as arginine and ornithine, which are crucial for maintaining cellular metabolic homeostasis. The presence of NAG suggests microbial adaptation to nitrogen-rich wastewater through enhanced amino acid synthesis. Similarly, L-glutamic acid, a central molecule in nitrogen metabolism, acts as an amino group donor in various transamination reactions. Its upregulation implies active nitrate assimilation, whereby nitrate is converted into amino acids such as alanine, aspartic acid, and serine (Fig 3c). Conversely, several metabolites were significantly downregulated, including Phe-Gly, Val-Leu, Ser-Phe, Glycylglycine, and 2-(Aminomethyl)phenol. Under anaerobic conditions, dipeptides such as Phe-Gly, Val-Leu, and Ser-Phe may serve as carbon and electron donors during microbial nitrate reduction. Branched-chain amino acids are particularly susceptible to catabolism, generating essential cofactors such as nicotinamide adenine dinucleotide (NADH) and nicotinamide adenine dinucleotide phosphate (NADPH) to drive microbial metabolism. The downregulation of Glycylglycine likely reflects its preferential utilization as a carbon or nitrogen source in nitrate-enriched environments. Meanwhile, 2-(Aminomethyl)phenol—an aromatic amine—can become protonated under acidic conditions, potentially affecting its intracellular stability and transport. Microorganisms may reduce the accumulation of such compounds to avoid cytotoxic effects.

### 3.3. Proteomic analysis

#### 3.3.1. Analysis of differentially expressed proteins (DEPs).

**Fig 4:**
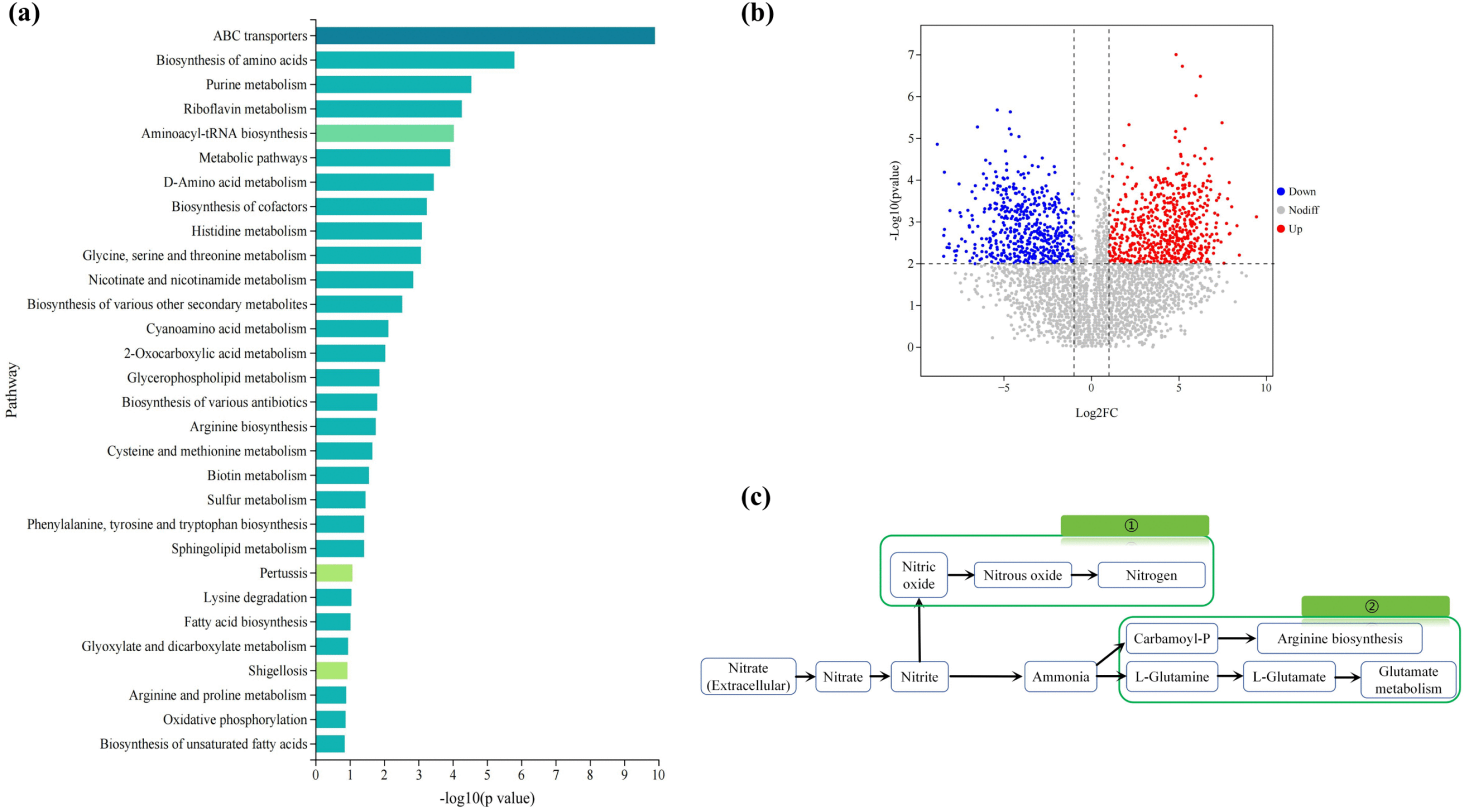
(a) Number of up- and down-regulated proteins of biological process (BP). (b) DEPs within the BP in the groups classified using the gene ontology (GO) database. (c) Pathway classification based on KEGG enrichment analysis of DEPs in the group.

The number of DEPs involved in the 120 mL dosing was 355. The number of downregulated proteins exceeded that of upregulated proteins (p < 0.05, Fig 4a).

DEPs are mainly categorized into BP, cellular component (CC), and molecular function (MF). BP is a key category of metabolic pathways, with several primary biological process groups identified, including peptide transport, the phosphoenolpyruvate-dependent sugar phosphotransferase system (PTS), signal transduction, and phage shock (Fig 4b). Peptide transport is essential for the transport of amino acids and small peptides, which are critical for microbial growth and metabolism and may indirectly influence the synthesis and function of Nar [34]. The PTS is an important mechanism in strain for sugar uptake, enabling the phosphorylation-driven transport and metabolism of carbon sources [35]. In anaerobic environments, PTS facilitates the efficient uptake and metabolism of sugars, such as glucose and fructose, to meet bacterial energy demands. The phage shock protein is a bacterial protective mechanism activated under conditions of external membrane stress [36]. During nitrate degradation, strain may encounter various forms of stress, including physical, chemical, or osmotic pressures, such as high concentrations of NO_3_^-^-N or its reduction products (like NO_2_^-^-N). This response helps strain maintain the efficient transport and metabolism of nitrate, even in the presence of acid stress, thereby enhancing their ability to degrade nitrate efficiently and improving their overall adaptive capacity to harsh environmental conditions. In conclusion, DEPs identified in this study are involved in crucial BP, including peptide translocation, the PTS, signaling, and the phage shock response. These processes are essential for bacterial growth, energy metabolism, and stress adaptation, particularly under conditions like nitrate degradation and acid stress. By supporting efficient sugar uptake, stress responses, and nitrate metabolism, these mechanisms work together to enhance the strain’s ability to degrade nitrate in challenging environments.

KEGG pathway analysis of the DEPs revealed significant enrichment. Pathways that were notably enriched include starch and sucrose metabolism, teichoic acid biosynthesis, and the PTS (p < 0.05, Fig 4c). These findings suggest that these pathways play important roles in the physiological processes, potentially contributing to energy provision, metabolic regulation, and the maintenance of cellular stability. The starch and sucrose metabolic pathways break down polysaccharides such as starch and sucrose into monosaccharides like glucose and fructose. These monosaccharides are further metabolized through the glycolytic pathway (glycolysis) and the TCA cycle to produce ATP and reducing equivalents (e.g., NADH) [37]. Starch and sucrose metabolism promotes the efficient degradation of nitrate by providing carbon sources, energy, and reducing power for nitrate reduction, while also supporting metabolic homeostasis and microbial community collaboration. This metabolic pathway is a critical source of both energy and materials necessary for nitrate degradation. Teichoic acid biosynthesis are important components of the cell wall of Gram-positive strain and are involved in cell wall stability and shape maintenance [38]. They are also implicated in extracellular ion exchange and the stability of membrane proteins. Cell wall stability helps maintain the function of nitrate transport proteins and related enzymes. The biosynthesis of teichoic acid helps the cell to resist environmental stresses under osmotic stresses that may be triggered by high concentrations of nitrate or nitrite. Sugar metabolites from the PTS system can provide energy and reducing equivalents for nitrate reduction through glycolysis and the TCA cycle [39]. Pantothenic acid (vitamin B5) is a precursor of coenzyme A (CoA), which is a key cofactor in many metabolic pathways (e.g., fatty acid metabolism, TCA cycle) [40]. Coenzyme A is involved in metabolic processes such as the TCA cycle and fatty acid oxidation to provide energy for nitrate reduction. Through CoA-related metabolic reactions, important biosynthetic precursors and signaling molecules are generated, contributing to the overall adaptive and degradative functions of the cell.

#### 3.3.2. Nitrogen metabolism.

**Fig 5:**
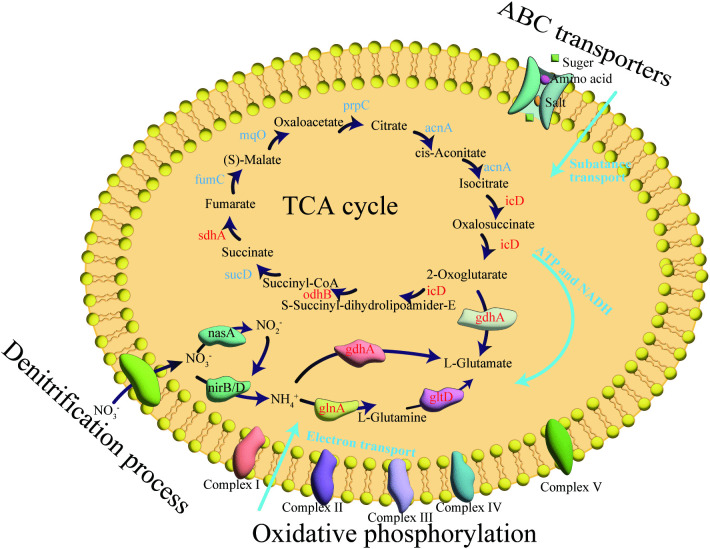
Nitrogen metabolism pathway and acid tolerance mechanism of strain ND7.

Unlike most reports, the reduction of nitrate by strain ND7 occurred through assimilation, rather than the conversion of nitrate to N_2_ or N_2_O. First, *nirB* and *nirD* in strain ND7 convert NO₃⁻ to NH₄⁺ (Fig 5). Ammonium is then assimilated through two metabolic pathways. One pathway involves the direct conversion of ammonia to L-glutamic acid (L-Glu), while the other converts ammonia to L-glutamine (L-Gln) [41]. The gene encoding glutamine synthetase, *glnA* (Log2FC: 0.33), was upregulated under high acid stress, promoting the accumulation of L-Gln, which is further converted to L-Glu via *gltD* (Log2FC: 0.25). L-Gln serves as an important energy source for cellular biosynthesis and metabolism [42], while L-Glu is a pivotal substance involved in protein and sugar metabolism. The accumulation of L-Gln and L-Glu may help strain ND7 resist high acidity, enabling it to effectively remove nitrate even under low pH conditions. Interestingly, the *nirK/S*, *norB*, and *nosZ* genes were not detected in strain ND7. Therefore, unlike other studies, when nitrate is converted to nitrite, ND7 instead converts it to ammonia, which is then synthesized into amino acids via genes such as *gdhA*, rather than reducing nitrite to gaseous products (NO, N₂O, N₂). Overall, all genes involved in the conversion to amino acids within the nitrogen metabolism pathway were significantly upregulated in strain ND7. This may be the primary reason for its ability to resist high acidity and effectively remove NO₃ ⁻ .[43].

#### 3.3.3. Other metabolisms.

*3.3.3.1. TCA cycle:* It has been suggested that the co-regulation of various metabolic pathways plays a key role in maintaining cellular homeostasis and enhancing cell survival under acid stress conditions [44]. Pathways such as central carbon metabolism, membrane transport, and electron transfer were important for strain to protect their cells from damage in highly acidic environments [45]. The TCA cycle is the central metabolic network of organisms, closely associated with the metabolic process of sugar, lipids, and proteins and it provided precursors for energy metabolism [46]. In this study, most of the differentially expressed genes (DEGs) in the TCA cycle (*icD*, *odhB*, *sdhA*) were significantly upregulated, suggesting that strain ND7 produced more energy during the degradation of nitrate in wastewater. Liu et al. [44] found that under acid stress, the enzyme activity in the TCA cycle of *Lactobacillus plantarum* 120 (Lp-120) increased. Increased enzyme activity could produce more energy for other cellular metabolic processes [47]. The above results suggest that the enhanced TCA cycle provides more energy to the strains under acidic conditions, thereby improving the survival of the microorganisms.

*3.3.3.2. Oxidative phosphorylation:* Under acidic conditions, the present study revealed a significant downregulation of genes involved in the electron transport pathway of oxidative phosphorylation. Similar downregulation of these genes has also been reported in *Escherichia coli* under acidic conditions [48]. Additionally, many microorganisms resist exogenous stress by reducing respiration and suppressing the expression of electron transport chain genes [49]. In strain ND7, genes in the electron transport chain were also downregulated. The differences in results can be attributed to several factors: first, the degradation pathway of strain ND7 differs from that of conventional strains, as strain ND7 primarily converts nitrate to amino acids. Second, under optimal conditions, the electron transport chain plays a more significant role in denitrification. However, under acidic conditions, environmental stress may exert a more prominent influence on denitrification. Therefore, although the downregulated genes in strain ND7 may reduce electron transfer in the nitrogen cycle, they contribute to enhanced acid resistance.

*3.3.3.3. ABC transporters:* ABC transporters may be crucial in microbial resistance to acidic wastewater. Studies showed that ABC transporters could import substances such as salt ions, carbohydrates, amino acids, etc. into cells under nutrient-limited conditions [50]. A total of 95 DEGs were found to be associated with ABC transporter proteins in this study, 57 of which were significantly upregulated. The upregulation of these genes may be linked to the enhanced transport of amino acids, urea, and carbohydrates by strain ND7 under acidic conditions, which in turn supports cellular metabolism and improves denitrification efficiency. Similar results were reported by Liu et al. [44] Based on these findings, it can be inferred that membrane transport plays a direct role in the acid tolerance of strain ND7 under acidic conditions.

The proteomic results clearly demonstrate that the regulation of nitrogen metabolism gene expression is crucial for efficient acid denitrification. However, other metabolic pathways also play essential roles in acid resistance. Specifically, acid stress inhibited the cellular electron transport chain, while enhanced nitrogen metabolism and ABC transport facilitated more efficient conversion of nitrate to amino acids. Although nitrogen metabolism is pivotal for nitrate removal, other metabolic pathways also contribute significantly to acid resistance. Thus, the mechanism underlying efficient nitrate removal by strain ND7 under acidic conditions involves a synergistic interaction of multiple metabolic pathways.

## 4. Conclusion

The acid-tolerant *Lysinibacillus sp.*ND7 strain was newly isolated from sludge. The strain ND7 demonstrated excellent nitrate removal (NO₃ ⁻ -N) even under acidic conditions (pH 3.1). Metabolomic and proteomic analyses revealed that ND7 resisted acid stress by synergistically assimilating nitrate into amino acids, along with the activation of genes related to other metabolic pathways. This study highlights the promising potential of ND7 for acidic wastewater treatment and provides new insights into the mechanism of nitrate removal under acidic conditions.

## Supporting information

S1 Fig Changes in NO_2_^-^-N content during the course of the reaction process.
(TIF)

S2 Fig Changes in NH_4_^+^ content during the course of the reaction process. (TIF)

S3 Fig Changes in NO_3_^⁻^ -N content following sterilisation treatment.
(TIF)

S1 Data Datasets used in the research—Compressed files archive. (ZIP)
